# The effect of high intensity interval training with beetroot (Beta vulgaris) juice supplementation on serotonin and dopamine receptors expression, anxiety and depression in middle-aged diabetic rats

**DOI:** 10.22038/AJP.2022.20895

**Published:** 2022

**Authors:** Parisa Ghanbari, Sanaz Khajehzadeh, Asieh Sayyed, Davood Raeisi, Omidreza Salehi

**Affiliations:** 1 *Department of Sport Physiology, Behbahan Branch, Islamic Azad University, Behbehan, Iran*; 2 *Department of Internal Medicine, Faculty of Medicine, Ahvaz Jundishapour University of Medical Sciences, Ahvaz, Iran*; 3 *Department of Physical Education and Sport Sciences, University of Kurdistan, Sanandaj, Iran*

**Keywords:** HIIT, Beta vulgaris, Serotonin receptor, Dopamine receptor, Aging, Diabetes

## Abstract

**Objective:**

The aim of this study was to evaluate the effect of four weeks of high intensity interval training (HIIT) with beetroot juice supplementation (BJ) on serotonin and dopamine receptors in hippocampal tissue, as well as anxiety and depression in middle-aged diabetic rats.

**Materials and Methods:**

In this experimental study, 28 diabetic female rats (55 mg/kg, induced by streptozotocin) aged 12-14 months, weighing 280-320 g, were divided into (1) diabetic control (DC), (2) BJ, (3) HIIT, and (4) HIIT+BJ groups. Also, 7 healthy rats were included in the healthy control (HC) group to evaluate the effect of diabetes induction on the research variables. HIIT was performed for four weeks, 4 sessions per week (70-95% of maximum speed at high intensities; 50-60% of maximum speed at low intensities). Also, BJ was fed daily to rats at a dose of 10 ml/kg.

**Results:**

Hippocampal expression of dopamine receptor-1 (*Dop.R*), *5-hydroxytryptamine receptor* (*5-HT. R*), open arm entry percentage (OAE%) and movement rate in the HIIT, BJ and HIIT+BJ groups were significantly higher than the DC group. In the HIIT+BJ group, open arm time percentage (OAT%) was higher than the DC group. Levels of *Dop.R* gene expression were more affected by HIIT, and levels of *5-HT. R* were more affected by BJ supplementation; also, HIIT+BJ had a synergistic effect on reducing anxiety and depression.

**Conclusion:**

Although HIIT was more effective than BJ and HIIT+BJ on *Dop.R* and BJ supplementation on *5-HT.R* and improved anxiety and depression, both of HIIT and BJ were complementary in improving dopamine and serotonin receptor-dependent anxiety and depression and enhanced each other's effects.

## Introduction

Today, more than 90% of the elderly have a metabolic disorder, especially diabetes (Amidei et al., 2021 ▶). According to studies, metabolic disorders increase synergistically with aging, rising the risk of impaired memory and learning, anxiety, depression and physical disabilities (Amidei et al., 2021 ▶; Zhong et al., 2021 ▶). Disorders of the nervous system can lead to increased activity of reactive oxygen and nitrogen species, disorders of the hypothalamic-pituitary-adrenal (HPA) axis, inactive lifestyle, and abnormal genetic mutations (Zhong et al., 2021 ▶). Depression and anxiety appear to be unavoidable diseases following impaired glucose metabolism (Kleinridders et al., 2015 ▶). Impaired insulin function and hyperglycemia in the central nervous system increase the enzymes that break down dopamine and serotonin in brain tissue (Kleinridders et al., 2015 ▶; Zou and Sun, 2021 ▶).

Generally, researchers believe that neurotransmitter receptors act at the first functional levels in response to neurotransmitters; therefore, destruction at the receptor levels leads to the dysfunction of the neurotransmitter and the nervous system (Hashemi et al., 2012).

According to the studies, different treatment methods have been introduced in this regard, but exercise seems to improve physical and psychological health by improving neurotrophin function and body metabolism (Hosseini et al., 2021 ▶). Long-term exercise with the modulation of metabolic hormones, improvement of HPA axis function, and restoring the balance of acetylcholine and gamma-aminobutyric acid (GABA) system, leads to a decrease in neuronal damage (Basso and Suzuki, 2017 ▶); it can also reduce anxiety and depression in diseases of the nervous system by increasing dopamine (Dop.R) and 5-hydroxytryptophan or serotonin receptors (5-HT.R) (Shenas et al., 2021 ▶). In this regard, resistance training improved dopamine and serotonin receptors in rats with Alzheimer's disease (Shenas et al., 2021 ▶); also, high intensity interval training (HIIT) improved mental health in elderly rats (Hosseini et al., 2021 ▶). In previous studies, the optimal effect of endurance training on neurotrophin levels (Salehi and Hoseini, 2017 ▶), metabolic indices (Hosseini et al., 2020 ▶) and motor balance (Hosseini et al., 2020 ▶) in animal models with metabolic disorders and nervous disorder, has been reported.

Most of these studies examined the effect of exercise training in the long term; therefore, due to the progressive damage following impaired glucose metabolism and nervous system disorders, the efficient courses with a shorter duration seems necessary. It seems that the use of herbs along with exercise training be an advantageous way to benefit more from exercise (Hosseini et al., 2021 ▶). Beetroot (*Beta vulgaris*) (BR) is an annual plant that due to its natural sugars, fiber, protein, vitamins, minerals and nitrate (NO_3_) has many metabolic effects and is used in the treatment of some diseases (Arazi and Eghbali, 2021 ▶). *In vivo *and *in vitro* studies have shown that nitrate in beetroot is a precursor to nitric oxide, and nitrate in beetroot increases the expression of neurotrophins, modulates noradrenaline, and regulates serotonin and dopamine in various parts of the brain such as the hypothalamus and hippocampus (Arazi and Eghbali, 2021 ▶). Kozlowska et al. in 2020 ▶ reported that beetroot consumption increased the maximum oxygen consumption, and improved the function of neurotransmitters and the metabolism of trained individuals (Kozlowska et al., 2020 ▶); in another study, acute BR supplementation modulated sympathetic tone and improved heart rate function (Notay et al., 2017 ▶). 

Due to the importance of shortening the duration of treatment of metabolic disorders and prevention of related disorders such as central nervous system disorders, the present study attempted to provide a new solution against anxiety and depression regarding physiological indicators related to it in brain tissue. Therefore, the aim of the present study was to investigate the effect of four weeks of HIIT with BR juice supplementation on anxiety, depression, and *5-HT.R* and *Dop.R* expression in the brain tissue of middle-aged diabetic rats.

## Materials and Methods

In this experimental study, 39 female rats with an age range of 12-14 months and a weight range of 280-320 g, were prepared from the Center for Breeding and Reproduction of Laboratory Animals, Islamic Azad University, Marvdasht Branch, and were maintained for one week for adaptation in the Animal Exercise Physiology Laboratory of this university. It is noteworthy that during the research period, the ethical principles of working with laboratory animals were observed according to the Helsinki Agreement. The study protocol was approved with the code of ethics No. IR.IAU.BEHBAHAN.REC.1401.004 by Islamic Azad University of Behbahan Branch; also, laboratory animals were maintained in standard conditions with a temperature of 22-24°C, relative humidity of 55-60%, in polycarbonate cages with autoclave capability in an environment with standard light-dark cycle (12 to 12), and *ad libitum* access to water and animal food. On the eighth day, following 12 hr of fasting, 32 rats underwent peritoneal injection of 55 mg/kg streptozotocin (STZ) (0.05 molar, pH 4.5) dissolved in citrate buffer (manufactured by Sigma Aldrich, USA). Then, 4 days after the injection, blood glucose levels of rats were measured by tail punching using a glucometer (Glucoard 01) and rats with glucose level above 300 mg/dl were identified as diabetics (Hosseini, hamzavi, 2020 ▶). It is noteworthy that two of these rats were excluded from the study due to lack of hyperglycemia and two of the rats died later due to damage caused by hyperglycemia and reaction to STZ toxicity. Subsequently, rats with diabetes were divided into four groups, including (1) diabetic control (n=7) (DC), (2) beetroot juice supplementation (BJ) (n=7), (3) high intensity interval training (HIIT) (n=7), (4) HIIT + BJ (n=7). In addition, 7 healthy rats were assigned to the healthy control (HC) group for assessment of the effect of diabetes induction on the research variables.

**HIIT Protocol**

To develop the HIIT protocol, the intensity of the training based on the maximum oxygen consumption (VO_2max_) was used. To measure the VO_2max_ of elderly rats, they initially ran for 5 min on a special rat treadmill at a speed of 6 m / min and a zero-degree slope, and then, the speed of the treadmill was increased 3 meter/min every 3 min until the animals became exhausted and could no longer continue the protocol. The criterion for reaching VO_2max_ was the inability of the rats to continue the training protocol by increasing the speed and hitting the end of the treadmill 3 times consecutively in one minute. Thus, using the running speed, the rats VO_2max_, was obtained (Li et al., 2018 ▶). The rats then ran at a speed of 5 to 10 meters per minute for 10 to 15 min to adapt to interval training for a week. The training groups were then trained 4 sessions per week for four weeks**.** The rats running on the treadmill lasted 44 min, including 6 min of warm-up (at a speed of 10 to 12 meters per minute), 5 bouts of 4-min high intensity interval training (70 to 95% of maximum speed) and 4 bouts of 3-min low intensity interval training (50 to 60% of maximum speed) and 6 min of cooling**.** In the HIIT protocol, the treadmill inclination was considered to be zero-degree (Faezi et al., 2020 ▶). 

**Beetroot juice supplementation**

First, a sufficient amount of native beetroot of approved species and genus was prepared from Marvdasht Agricultural Jihad Center. Then, to prepare fresh beet juice, the beetroot was washed and chopped. Next, the fresh beet juice was extracted in a completely sterile environment and after passing through a paper strainer, 10 ml of fresh beet juice per kg of body weight was given to rats orally on the same day (Rabeh, 2015 ▶). Due to the possible damage of gavage and injection, in four weeks of the protocol, beetroot was supplemented to rats using small syringes (Nouri et al., 2021 ▶). 

For supplementation, 3 ml of the beetroot was poured into a small bottle and fed to each rat individually, based on the mean body weight of the rats.

**Assessment of anxiety-like behaviors**

Elevated plus-maze was used to assess anxiety-like behaviors. Elevated plus-maze was made of wood and had four arms in the form of a positive sign +; it also had open and close 10×50 cm arms, and both sides and end of the closed arms had walls of 40 cm high. The four arms led to the central area measuring 10×10 cm. The maze was placed by bases at a height of 50 cm above the ground.

The rats were placed in the central area of the maze, facing an open arm. During the 5 min that the animal moved freely in different parts of the maze, the open arm entry (OAE) and closed arm entry (CAE), the open arm time (OAT) and close arm time (CAT) were recorded. By open arm entry (OAE) and closed arm entry (CAE), it was meant that all four legs of the animal were in the arm. The time spent in each arm was calculated accordingly. Then, open arm time percentage (OAT%) and open arm entry percentage (OAE%) were measured using the following formula (Hosseini et al., 2021 ▶):

OAT%=OAT/(CAT+OAT)×100

OAE%=OAE/(CAE+OAE)×100

**Forced swimming test to assess depression**

Forced swimming test was used to measure depression. The forced swimming test is one of the most valid and common tests to measure rodent depression. This test lasts for 5 min and the rat behavior is recorded during this period. Conventionally, the cessation of the movement of the rat's limbs and its floating are considered immobility and the duration is considered the time of immobility. The animal was immersed in water for 15 min 24 hr before the test, to gain the experience of forced swimming (Hosseini et al., 2021 ▶). 

**Dissection and sampling procedure**

Forty-eight hours after the last training and supplementation session and following 12-hr of fasting, the rats were anesthetized by a combination (55 mg/kg) of ketamine and xylazine (15 mg/kg) made by Alfasan Co., the Netherlands. After ensuring anesthesia and analgesia in rats and fixing them, 15 ml normal saline was perfused to drain the brain tissues of blood and then after splitting the upper part of the skull and extracting the tissues, the hippocampal tissues of brain were carefully isolated and after weighing and washing were transferred to tissue-specific microtubes. To prevent damage due to rapid freezing of tissues, they were first stored at -20°C for six hours and were then transferred to -70°C; afterward, they were transferred to a molecular cell laboratory as soon as possible to evaluate physiological variables.

**Serotonin and dopamine receptors expression measurement procedure**

In this study, levels of serotonin and dopamine receptor gene expression were measured using qReal Time PCR. First, 50 mg of tissue was isolated from the hippocampus and incubated in medium, then the homogenized tissue was used to extract RNA. To extract RNA, the protocol of the manufacturer (QIAGEN Germany) was used; also, to ensure the quality of RNA, agarose gel electrophoresis together with absorbing light property at a wavelength of 260 nm was used with Sigma PicoDrop device (USA). Then, following cDNA synthesis, reverse transcription reaction was performed at the PUBMED site based on the guidelines of *5-HT.R* and *Dop.R* genes, using the protocol of the manufacturer in the fermentase kit (K1621) and the designed primers (Table 1). To determine the efficiency and specificity of the primers, the primers were evaluated using the software available on the NCBI site. Also, to measure the gene expression levels of the research variables, the *B2m* internal control gene was used. 2^-ΔΔCT ^formula was used to quantify the ratio of the desired gene to the reference gene.

**Data analysis procedure**

Parametric tests were used to analyze the data due to the normal distribution of findings. One-way analysis of variance was used to examine the differences among the groups and Tukey’s *post hoc* test was used to determine the differences among the groups. The data of the present study were analyzed using GraphPad Prism 8.3.3 software. Also, a significant level was considered for all analyzes (p≥0.05).

## Results

The results of one-way analysis of variance showed that there was a significant difference in the levels of *Dop.R* (p=0.001) and *5-HT.R* (p=0.001) gene expression in hippocampal tissue; also, the results showed that there was a significant difference in the OAT% (p=0.001), OAE% (p=0.001) and the mobility in the forced swimming test (p=0.001) in the research groups. 

**Figure 1:**
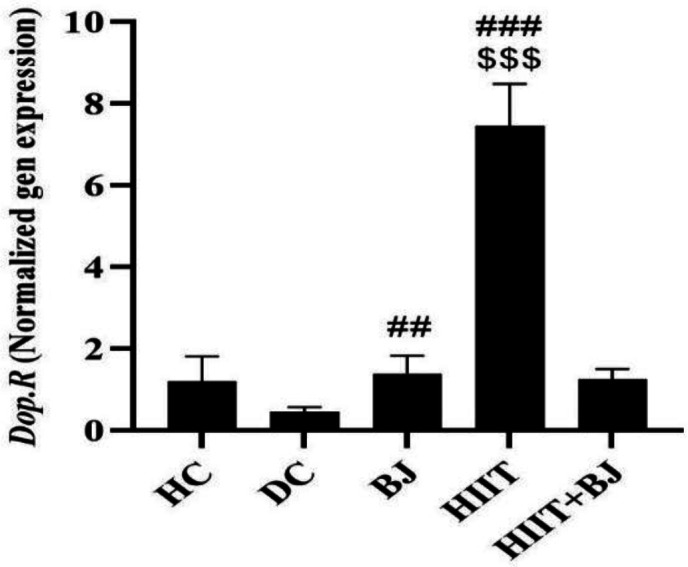
Levels of *Dopamine Receptor* gene expression in the hippocampal tissue of rats in different groups. ###(p≥0.001), and ##(p≥0.01) show significant increases in the Beetroot juice and High intensity interval training groups compared to the diabetic control group. $$$ (p≥0.001) significant increases compared to the Beetroot juice and High intensity interval training+ Beetroot juice groups

The results of Tukey’s *post hoc* test showed that there was no significant difference in the levels of *Dop.R* expression between the HC group and the DC group (p=0.13); however, levels of *Dop.R* expression in the BJ (p=0.03) and HIIT (p=0.001) groups were significantly higher than the DC group. Also, levels of expression in the HIIT group, were higher than the BJ (p=0.001) and HIIT + BJ (p=0.001) groups (Figure 1).

HC: Heathy control, DC: Diabetic control, BJ: beetroot juice supplementation, HIIT: high intensity interval training, HIIT+BJ: beetroot juice supplementation+ high intensity interval training

**Figure 2:**
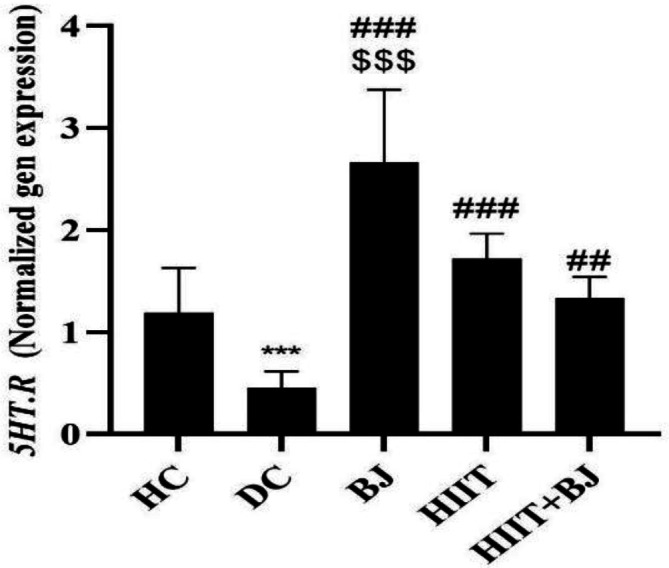
Levels of *5-hydroxytryptamine receptor *gene expression in the hippocampal tissue of rats in different groups.***(p≥0.001) significant decreases in the diabetic control group compared to the healthy control group.### (p≥0.001) and ##(p≥0.01) show significant increases in the Beetroot juice, High intensity interval training and High intensity interval training+ Beetroot juice groups compared to the diabetic control group. $$$ (p≥0.001) shows significant increases in the Beetroot juice group compared to the High intensity interval training and High intensity interval training+ Beetroot juice groups. HC: Heathy control, DC: Diabetic control, BJ: beetroot juice supplementation, HIIT: high intensity interval training, HIIT+BJ: beetroot juice supplementation+ high intensity interval training

The *5HT.R* gene expression in hippocampal tissue in the DC group was significantly lower than the HC group (p=0.01); but in the BJ (p=0.001), HIIT (p=0.001) and HIIT + BJ (p=0.002) groups, its expression was significantly higher than the DC group. Also, *5HT.R* gene expression in the BJ group, was significantly higher than the HIIT (p=0.001) and HIIT + BJ (p=0.001) groups (Figure 2). 

**Figure 3:**
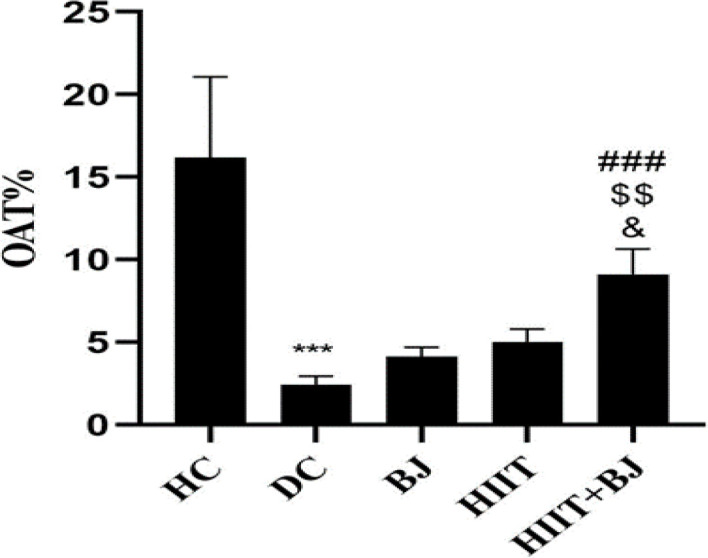
Levels of open arm time percentage (OAT%) in different groups. *** (p≥0.001) shows significant decreases in the diabetic control group compared to the healthy control group. ### (p≥0.001) shows significant increases in the High intensity interval training+ Beetroot juice group compared to the diabetic control group. $$ (p≥0.01) shows significant increases in the High intensity interval training+ Beetroot juice group compared to the Beetroot juice group. & (p≥0.05) shows significant increases in the High intensity interval training+ Beetroot juice group compared to the High intensity interval training group. HC: Heathy control, DC: Diabetic control, BJ: beetroot juice supplementation, HIIT: high intensity interval training, HIIT+BJ: beetroot juice supplementation+ high intensity interval training

**Figure 4:**
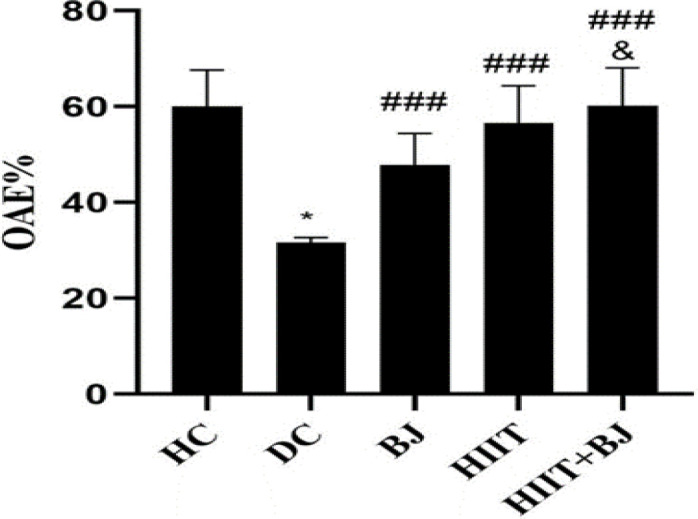
Levels of open arm entry percentage (OAE%) in different group *(p≥0.05) shows significant decreases in the diabetic control group compared to the healthy control group. ### (p≥0.001) shows significant increases in the Beetroot juice, High intensity interval training and High intensity interval training + Beetroot juice groups compared to the diabetic control group. & (p≥0.05) shows significant increases in the High intensity interval training+ Beetroot juice group compared to the Beetroot juice group. HC: Heathy control, DC: Diabetic control, BJ: beetroot juice supplementation, HIIT: high intensity interval training, HIIT+BJ: beetroot juice supplementation+ high intensity interval training

Levels of OAT% in the HC group were significantly higher than the DC group (p=0.001); also, in the BJ (p=0.65) and HIIT (p=0.27) groups, there were no significant difference compared to the DC group, but OAT% in the HIIT + BJ group was significantly higher than the DC (p=0.001), BJ (p=0.003) and HIIT (p=0.02) groups (Figure 3). Levels of OAE% in the HC group were significantly higher than the DC group (p=0.016), but in the BJ (p=0.001), HIIT (p=0.001) and HIIT + BJ (p=0.001) groups, were significantly higher than the DC group. Also, in the HIIT + BJ group, OAE% was significantly higher than the BJ group (p=0.014) (Figure 4).

**Figure 5:**
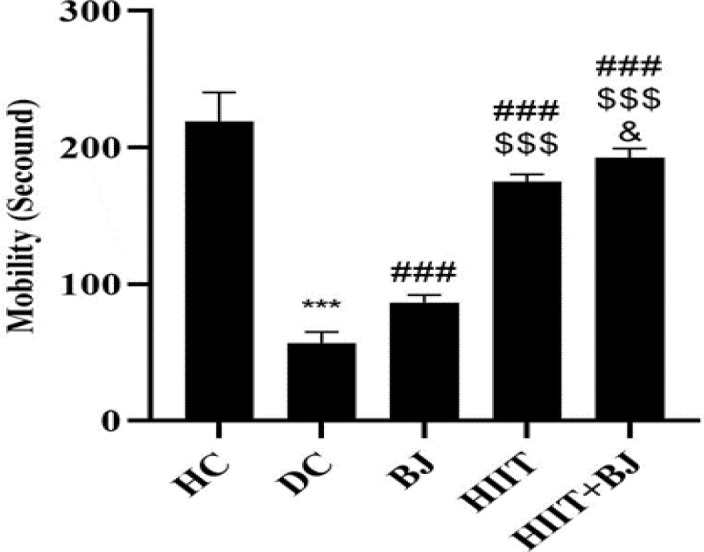
Levels of movement in the forced swimming test in different groups.***(p≥0.001) shows significant decreases in the diabetic control group compared to the healthy control group. ###(p≥0.001) shows significant increases in the Beetroot juice, High intensity interval training and High intensity interval training+ Beetroot juice groups compared to the diabetic control group.$$$ (p≥0.001) shows significant increases in the High intensity interval training and High intensity interval training+ Beetroot juice groups compared to the Beetroot juice group. & (p≥0.05) shows significant increases in the High intensity interval training+ Beetroot juice group compared to the High intensity interval training group. HC: Heathy control, DC: Diabetic control, BJ: beetroot juice supplementation, HIIT: high intensity interval training, HIIT+BJ: beetroot juice supplementation+ high intensity interval training

Levels of mobility in the DC group were significantly lower than the HC group (p=0.001), but in the BJ (p=0.001), HIIT (p=0.001) and HIIT + BJ (p=0.001) groups, the levels were significantly higher than the DC group. Also, in the HIIT group (p=0.001) and HIIT + BJ (p=0.001) groups, the levels were significantly higher than the BJ group. Besides, in the HIIT + BJ group, the levels were significantly higher than the HIIT group (p=0.04) (Figure 5).

## Discussion

The results of the present study showed that HIIT increased the levels of *Dop.R* and *5HT.R* expression in hippocampal tissue and increased OAE% and movement in the forced swimming test in middle-aged rats with diabetes. Aging, especially in women, is known with hormonal disorders, and changes in 17β-estradiol levels in these people are associated with increased oxidative stress, vasomotor system disorders, osteoporosis, chronic diseases and the risk of diabetes (Anklam et al., 2021 ▶).

In other words, the decrease in estrogen is associated with a decrease in the ability of the nerve cell to metabolize its main fuel, glucose, and disrupts energy supply systems such as glycolysis, citric acid cycle, and aerobic and anaerobic metabolism, as well as the electron transfer chain. Simultaneously, metabolic defects in the brain enhance metabolic disorders by increasing oxidative stress, apoptosis, amyloid beta accumulation, and finally disorders of the nervous system in the elderly (Hugenschmidt et al., 2021 ▶). 

Studies have shown that increased oxidative stress disrupts protein levels and DNA transcription, and even destroys the receptors of these neurotransmitters at the cell levels, and eventually, leads to cognitive impairment (Hashemi et al., 2012; Shenas et al., 2021 ▶).

In the pathophysiology of anxiety and depression following metabolic disorders in aging, it appears that impaired glucose and insulin metabolism may lead to changes in cortisol. Increased cortisol due to disruption of the HPA axis is associated with disruption of various neurotransmitters such as norepinephrine, serotonin, and dopamine. In addition, following diabetes, dysfunction of the intestinal-cerebral axis is associated with disorders of hormones such as leptin, ghrelin, adiponectin, and melatonin, which leads to an increase in inflammatory factors in brain tissue, and ultimately, damage to the serotonergic, dopaminergic, and orexinergic systems can lead to anxiety and depression (Subba et al., 2021 ▶). On the other hand, exercise can affect the health of the brain by improving the metabolism of energy substrates, increasing neurotrophins, and increasing neuroplasticity. In other words, sports activities have a mechanism related to fatigue, so that the dopaminergic system becomes more active when you start exercising, as lower levels of dopamine are associated with fatigue. It also appears that with starting exercise, the dopaminergic system by increasing the transcription pathways of genes, augments the expression of type 2 and 3 dopamine receptors, enhances dopamine secretion, and even attract blocked dopamine in other parts of the brain to cope with fatigue (Marques et al., 2021 ▶). In addition, exercise can increase the expression of dopamine and serotonin receptors and reduce anxiety and depression by increasing the transcription of genes, reducing oxidative stress, increasing levels of neurotrophins, ephedrines, and brain-derived neurotrophic factor (BDNF), improving the sensitivity of neurotransmitter receptors in the synaptic space, neurogenesis, endorphins secretion, dopamine transporters, serotonin and modulation of tyrosine hydroxylase activity (Shenas et al., 2021 ▶). Still, serotonin metabolism appears to be different in various parts of the brain. In addition, serotonin changes following exercise activities depend on intensity, duration of training, and measured portion of serotonin levels; as a result, an increase in serotonin in the hypothalamus, medulla oblongata and brainstem following exercise was reported, but a decrease in serotonin in hippocampal tissue was also reported in a study (Heijnen et al., 2016 ▶). Hence, the greater effect of HIIT on dopamine following exercise can be attributed to this mechanism. Consistent with the present study, it was shown that eight weeks of resistance training increased the expression of serotonin and dopamine receptors in the hippocampal tissue of rats with Alzheimer's disease (Shenas et al., 2021 ▶). In another study, resistance training increased beta-endorphin and dopamine levels, while there was no significant change in serotonin or tryptophan levels after exercise in untrained men (Moghadam et al., 2021 ▶). On the other hand, 24 weeks of resistance training significantly reduced serum levels of serotonin, dopamine, epinephrine and norepinephrine in elderly women (Kim et al., 2019 ▶); it seems that the difference between the results of this study and the present study is due to differences in the method of measurement and intensity of training.

The results of the present study showed that BJ supplementation increased the expression of *Dop.R* and *5HT.R* in hippocampal tissue, and augmented OAE% and the movement of forced swimming test in middle-aged diabetic rats. Studies showed that BJ has multifaceted biological activities due to the presence of beta alanines (beta cyanins and beta xanthines), flavonoids, polyphenols, saponins, and nitrates, as well as the richness in minerals such as potassium, sodium, phosphorus and calcium. In other words, this medicinal plant has enzymatic and non-enzymatic antioxidant effects and can play a role in improving cell function by different mechanisms. This medicinal plant also leads to transcription of metabolic genes by activating the AMP-activated protein kinase signaling pathway; BJ can also initially lower blood glucose levels by activating certain hormones and inhibiting alpha-amylase, alpha-glucosidase, and increasing paraoxinase. Changes in cortisol following BJ administration also appear to increase gluconeogenesis by acting on adrenocorticotropin (ACTH) (Mirmiran et al., 2020 ▶). Subsequently, further increase in cortisol levels leads to an increase in tryptophan analyzing enzyme and an increase in serotonin (Mirmiran et al., 2020 ▶; Subba et al., 2021 ▶). In a study, researchers found that BJ triggers intracellular signals by increasing nitric oxide synthase and, at the same time, enhancing cerebral blood flow, increases neurotrophins, which have antidepressant effects. Besides, BJ increased dopamine and serotonin expression in brain tissue by modulating tyrosine and cAMP/NRF signaling (Kozlowska et al., 2020 ▶). In addition, in a study, researchers showed that BJ consumption was associated with improved nervous system function in obese men and women (Babateen et al., 2020 ▶). Considering the role of beetroot in enhancing cortisol and its effect on HPA axis, it seems that the greater effect of BJ on increasing serotonin receptor compared to HIIT+BJ and HIIT depends on this cellular pathway. 

The results of the present study showed that HIIT with BJ supplementation increased *5HT.R* expression in hippocampal tissue, OAT% and OAE%, and enhanced movement in the forced swimming test in diabetic rats. In addition, levels of *Dop.R* gene expression were more affected by HIIT and levels of *5HT.R* gene expression by BJ supplementation, while HIIT + BJ had a synergistic effect on reducing anxiety and depression. 

Studies have shown that exercise training, depending on the intensity, type and duration, improves neuronal metabolism, increases neurotrophins, and facilitates transcription pathways of type 2 and 3 dopamine receptors expression (Marques et al., 2021 ▶). An increase in ephedrines, BDNF, dopamine transporters, and serotonin results in increased expression of dopamine and serotonin receptors and decreased anxiety and depression (Heijnen et al., 2016 ▶; Shenas et al., 2021 ▶). However, supplementation of beetroot juice with the mechanism of improving the antioxidant enzymes and non-enzymatic antioxidants, activation of AMPK, altering cortisol levels in the direction of serotonin synthesis (Mirmiran et al., 2020 ▶; Subba et al., 2021 ▶) has a favorable effect on depression and anxiety. Nonetheless, the present study showed that HIIT with greater effect than BJ and HIIT+BJ on dopamine improved anxiety and depression in diabetic rats, while beetroot juice supplementation with a more favorable effect on serotonin had anti-anxiety and anti-depressant effects.

In addition, according to the results, beetroot seems to increase catecholamine hormones by increasing cortisol levels and affecting sympathetic nerve tone, which may be a justification for the moderating effect of beetroot in interaction with HIIT (Mirmiran et al., 2020 ▶; Subba et al., 2021 ▶).

In this regard, a study showed that supplementation of beetroot juice three hours before exercise training could modulate the HPA-responsive response and improve the sympathetic nervous system (Arazi and Eghbali, 2021 ▶) . Also, in another study, consumption of BJ at doses of 400 and 800 mg nitrate improved reaction, cognitive function, and sympathetic and parasympathetic tone in male athletes (Miraftabi et al., 2021 ▶). In addition, six weeks of exercise training and BJ supplementation enhanced metabolic function and improved motor and pre-motor cortex in the elderly (Petrie et al., 2017 ▶). Based on the research, it seems that the lack of evaluation and measurement of serotonin and dopamine receptors following the interventions of this study is one of the limitations of this study. Therefore, it is suggested that in future studies, cell pathways and different subunits of these two receptors be evaluated. Also, given the role of glucose metabolism and contradictory changes in serotonin following diabetes induction, it seems that the lack of serotonin-dependent metabolic markers in different parts of the brain is one of the limitations of the present study. Therefore, it is suggested that future studies examine the changes in these two receptors with emphasis on metabolic pathways. Another limitation of this study is the lack of control of the amount of BJ drinking, so it is suggested that the exact amount of BJ consumption be determined for each rat in the next studies.

Although HIIT had a greater effect on dopamine and BJ supplementation had a greater effect on serotonin in improving anxiety and depression, both interventions could complement each other in the path of dopamine and serotonin receptor-dependent anxiety and depression, and reduced anxiety and depression in middle-aged diabetic rats.

## Conflicts of interest

The authors have declared that there is no conflict of interest.
